# The Edge Stresses and Phase Transitions for Magnetic BN Zigzag Nanoribbons

**DOI:** 10.1038/s41598-017-08364-5

**Published:** 2017-08-10

**Authors:** Junkai Deng, Yuefeng Yin, Huanhuan Niu, Xiangdong Ding, Jun Sun, Nikhil V. Medhekar

**Affiliations:** 1 State Key Laboratory for Mechanical Behavior of Materials, Xi’an Jiaotong University, Xi’an, 710049 China; 2 Department of Materials Science and Engineering, Monash University, Wellington Road, Victoria, 3800 Australia

## Abstract

The edge states are of particular importance to understand fundamental properties of finite two-dimensional (2D) crystals. Based on first-principles calculations, we investigated on the bare zigzag boron nitride nanoribbons (zzBNNRs) with different spin-polarized states well localized at and extended along their edges. Our calculations examined the edge stress, which is sensitively dependent on the magnetic edge states, for either B-terminated edge or N-terminated edge. Moreover, we revealed that different magnetic configurations lead to a rich spectrum of electronic behaviors at edges. Using an uniaxial tensile strain, we proposed the magnetic phase transitions and thereby obtained the metallic to half-metallic (or reverse) phase transitions at edges. It suggests zzBNNR as a promising candidate for potential applications of non-metal spintronic devices.

## Introduction

Two-dimensional (2D) materials, such as graphene, hexagonal boron nitride (h-BN) and transition metal dichalcogenides, have attracted significant attentions in characterizing their unique properties as well as their crystalline defects^1–3^. In particular, the edge states in 2D monolayers are found to be extremely important in the design of 2D material-based devices, attributed to their physical, chemical, and electronic properties that are distinct from their intrinsic counterpart^4–13^. Recent experiments and theoretical calculations have revealed that the edge stress can play a dominant role in influencing the intrinsic topology of the monolayer, which can potentially be used to tune the electronic and chemical properties of the edges and their vicinity^14–18^. This has sparked growing interests in understanding the relationship between mechanical, electronic and magnetic properties of stable edges for 2D materials.

Monolayer h-BN provides one of the simplistic examples of multicomponent 2D materials^19^. When further constraining h-BN to one-dimensional nanoribbon, many interesting physical phenomena and potential applications can be expected due to its reactive edges^20–22^. It is reported that the reactive edges of h-BN are prone to intrinsic reconstruction, or extrinsic passivation by functional groups such as hydrogen atoms^23, 24^. However, some recent experiments have suggested efficient approaches of synthesizing h-BN nanoribbons with bare edges^25, 26^. Interestingly, a stoichiometric zigzag BN nanoribbon (zzBNNR) involving bare boron-terminated and bare nitrogen-terminated zigzag edges can exist in either antiferromagnetic or ferromagnetic state^27–29^. And in both of the states, individual boron or nitrogen edges can reach a variety of localized magnetic patterns^27–29^. These distinct edge magnetic states in principle originated from different electronic structures of the nanoribbon. Since the edge stress of nanoribbon is also intimately linked with the underlying electronic structure, different magnetic configuration at edge can be expected to lead to different edge stress states, and thereby affecting the topology of the near-edge region of the BN nanoribbon.

In this paper, using first-principles calculations, we determine the distinct edge stresses for non-equivalent individual edges with different magnetic configurations for zzBNNRs. We find all edges possess compressive stress except for B-terminated edge with antiferromagnetic ordering. It demonstrates that the magnetic behavior of zzBNNRs is closely related with the stress state as well as electronic structure at the edges. Furthermore, via strain engineering, we show that the edge magnetic configuration of zzBNNR can be selectively changed by applying a uniaxial elastic strain. If the tensile strain exceeds a certain threshold, the magnetic states at the edges will undergo a spontaneous change to produce a metallic 〈–〉 half-metallic transition at B and N edges. Our results suggest the potential for utilizing strain-induced magnetic transitions in the design of spintronics devices based on zzBNNRs.

## Results and Discussions

**Figure 1:**
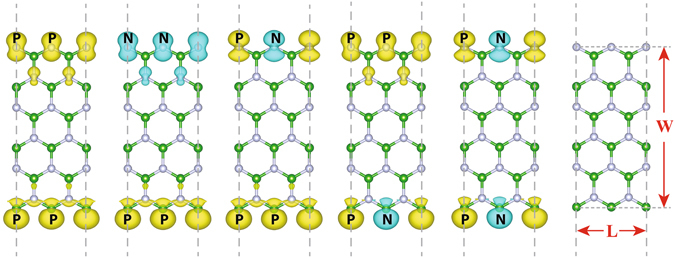
Schematic representations of the five edge magnetic configurations studied for stoichiometric zigzag BN nanoribbons. Each state is denoted as a combination of two pairs of positive (**p**) and negative (**n**) spin moments, according to the magnetization on the B and N edges, respectively.

First, we examine the stability of different spin polarized configurations at the strain-free state. Five basic non-equivalent spin arrangements are considered based on the structure as shown in Fig. 1, namely B(pp)/N(pp), B(pp)/N(nn), B(pp)/N(pn), B(pn)/N(pp) and B(pn)/N(pn), where p and n denote positive (spin-up) and negative (spin-down) magnetic moment, respectively. The five magnetic configurations correspond to coupling of ferromagnetic arrangements (B(pp)/N(pp) and B(pp)/N(nn)), antiferromagnetic arrangements (B(pn)/N(pn)) as well as coupling of different magnetic arrangements (B(pp)/N(pn) and B(pn)/N(pp)) at each edge. We also calculate a non-spin case for reference. We find that every magnetic pattern remains stable with the changing width of zzBNNRs. Table 1 presents the averaged energies of different magnetic configurations with respect to the ground state for all widths considered (See details in Methods). Our results show that for all the zzBNNRs studied, all spin polarized configurations are energetically favorable (about 300 meV per edge atom energy lower) than the non-spin case. The B(pn)/N(pp) configuration is found to be the ground state solution. All other spin polarized states have relative close energy (within 31 meV per edge atom) with the ground state, indicating that they are all thermally accessible at ambient conditions. These findings are consistent with previous theoretical studies on the magnetic states of zzBNNRs^27, 28^. In addition, it is noted that B(pp)/N(pp) and B(pp)/N(nn) has almost the same energy, demonstrating the absence of edge-to-edge coupling between magnetic orderings.

**Table 1:** Averaged energies of different edge magnetic configuration of zzBNNRs relative to the ground state configuration.

Magnetic configuration	Relative energy (meV/edge atom)
B(pp)/N(pp)	5.71
B(pp)/N(nn)	5.70
B(pp)/N(pn)	30.50
B(pn)/N(pp)	0
B(pn)/N(pn)	17.03

**Figure 2:**
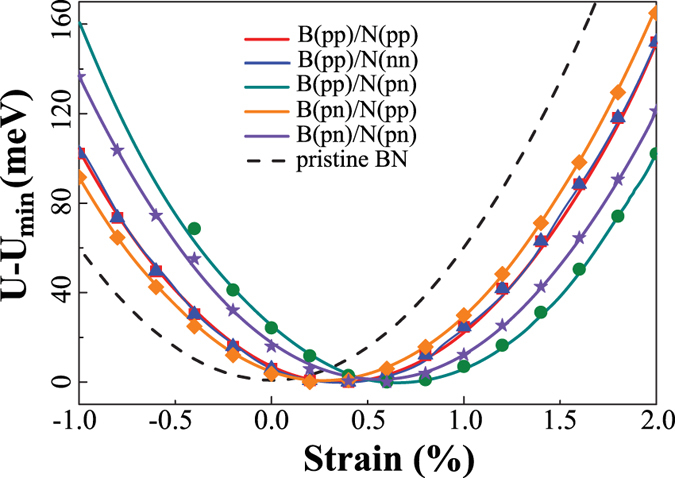
Variation in potential energy with applied strain for zzBNNRs with different magnetic configurations and pristine BN.

Next we evaluate the edge stress for different magnetic configurations of zzBNNRs shown in Fig. 1. To calculate the edge stress, we apply uniaxial strains on zzBNNRs. Figure 2 shows the variation in relative potential energy change (*U* − *U*_*min*_) of zzBNNRs of an width of 1.16 nm with different spin arrangements as a function of the uniaxial strain. *U*_*min*_ refers to the potential energy minimum at a certain magnetic state. Compared with the pristine monolayer h-BN, the potential energy has a minimum at a non-zero strain called the residual strain *ε*. The residual strain varies clearly for different edge spin states and follows the order: B(pp)/N(pn) > B(pn)/N(pn) > B(pp)/N(nn) ~ B(pp)/N(pp) > B(pn)/N(pp). Our further calculations show that the width of zzBNNRs has a size effect on the magnitude of the residual strain, but the general trend remains unchanged as the width increases.

The edge mechanical properties of a two-dimensional monolayer can be derived from the established model of surface stress of a three-dimensional crystal^30, 31^. Following this approach, The edge stress can be extracted by fitting the curve in Fig. 2 using the following equation:

1 $$U-{U}_{min}=\frac{1}{2}ELW{\varepsilon }^{2}+{E}_{s}L{\varepsilon }^{2}+\tau L\varepsilon ,$$

where *τ*, *L*, *W*, *E* and *E*_*s*_ denote the edge stress, the length of the nanoribbon, the width of the nanoribbon, bulk elastic modulus and edge elastic modulus for two-dimensional crystal, respectively. The first two terms are only related with the edge termination and should be identical for all bare zzBNNRs considered in this study. The third term with the edge stress is a deciding factor that leads to different potential curves for different magnetic configurations in Fig. 2.

**Figure 3:**
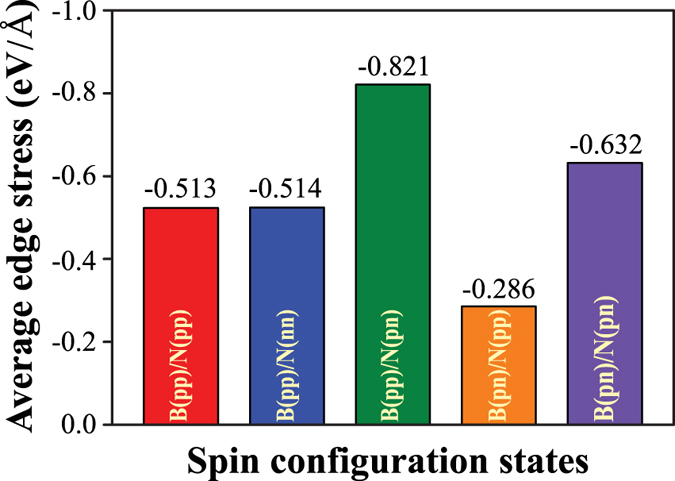
Averaged edge stress of zzBNNRs with different magnetic configurations.

Figure 3 presents the averaged edge stresses of zzBNNRs with different magnetic configurations for all widths considered. All zzBNNRs show compressive average edge stresses. It is clear that the edge stress is significantly influenced by magnetic states at the edges. The highest edge stress is found for B(pp)/N(pn) (−0.821 eV/Å), while the lowest is found for B(pn)/N(pp) (−0.286 eV/Å). The order of the magnitude of edge stress of different magnetic configurations is found to be the same as their relative energies as shown in Table 1.

To further analyze the effect of different magnetic patterns on the edge stress, we calculate the edge stresses for the individual edges based on the triangular hole model established in our previous work^31^. We obtain the edge stress for B(pp) by constructing a triangular hole terminated with identical edges inside a BN monolayer. All other individual edge stresses can be derived through the correlation between the total edge stress of nanoribbon and individual edge stresses of two specific edges, i.e. *τ*(zzBNNR) = *τ*(N-edge) + *τ*(B-edge). The results are listed in Table 2. We find that except for B(pn), all other edges are under compressive stress. For B-terminated edges, the magnetic ordering has a significant effect on the edge stress state. B(pp) and B(pn) have almost the same magnitude of edge stress, but opposite stress states. N-terminated edges bear larger edge stresses than B-terminated edges, with N(pn) having the largest edge stress of 1.42 eV/Å.

**Table 2:** The averaged edge stresses (*τ*) of individual edges of zzBNNRs.

Magnetic configuration	Edge stress (eV/Å)
B(pp)	−0.22
B(pn)	0.21 ± 0.05
N(pp)/N(nn)	−0.83/−0.84
N(pn)	−1.42

**Figure 4:**
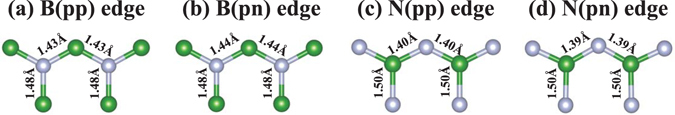
Bonding profile at the edges for different spin configurations.

We also measure the bond lengths at edges with different magnetic patterns as shown in Fig. 4. Keeping the width of zzBNNR as the value of pristine monolayer, the edge B-N bond lengths show clear difference after structural relaxation. The B-N bond lengths at B-terminated edges (1.43 Å for B(pp) and 1.44 Å for B(pn)) remain very similar to that in bulk (1.46 Å), leading to smaller edge stresses. However, at N-terminated edges, significant changes in B-N bond lengths are observed. The relaxed outermost edge B-N bond lengths (1.40 Å for N(pp) or N(nn) edge, and 1.39 Å for (N(pn) edge) are much shorter than that in bulk, indicating that the edge atoms at N-terminated edges “feel” a stronger intrinsic compressive stress. Increasing the width of zzBNNR (W) can relax such compressive stress and thereby lower its potential energy, which is in good agreement with the results shown in Fig. 2.

**Figure 5:**
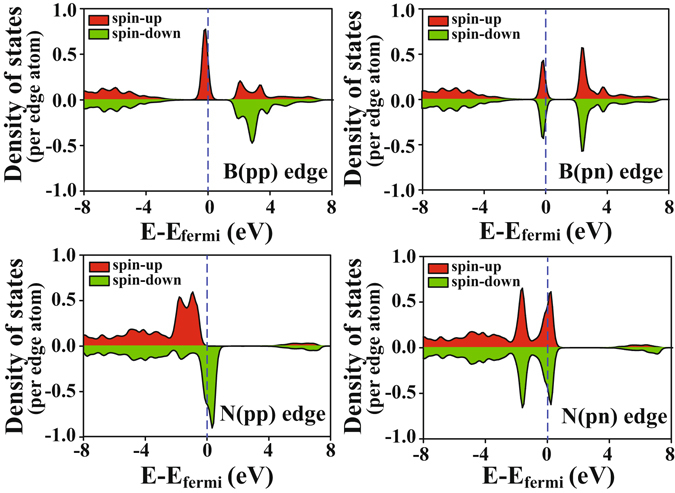
Spin polarized PDOS of individual edges of zzBNNRs.

In order to investigate the origin of the difference of edge stress states between B-terminated edges and N-terminated edges under the influence of magnetic patterns, we calculate the spin-polarized projected density of states (PDOS) of individual edges as shown in Fig. 5. It can be seen that localized midgap states, shown as spikes near the Fermi level, are only found in B-terminated edges with ferromagnetic ordering (B(pp)). When the magnetic configuration turns to antiferromagnetic (B(pn)), the midgap peak is split into two separate small peaks in both spin-up and spin-down channel. For N-terminated edges, larger density of states appears within the nearby regime of Fermi level. We linked the localized electronic structure with edge stress state by calculating the center of electronic states near the Fermi level (*E*_*avr*_) by^32^:

2 $${E}_{avr}=\frac{\int E({N}^{+}(E)-{N}^{-}(E))dE}{\int ({N}^{+}(E)-{N}^{-}(E))dE}-{E}_{F}$$

The position of the states can tell the bonding strength of B-N at the edges. We obtained *E*_*avr*_ for B(pp), B(pn), N(pp) and N(pn) are −0.452 eV, −0.453 eV, −1.317 eV and −1.415 eV, respectively. N-terminated edges show significant downshift of electronic states compared to B-terminated edges. It suggests that the B-N bonding strength at N-terminated edges is much stronger than that at B-terminated edges, providing an explanation for much shorter B-N bond lengths at N-terminated edges in Fig. 4. The order of downshift also reflects the magnitude of edge stress at individual edges.

**Figure 6:**
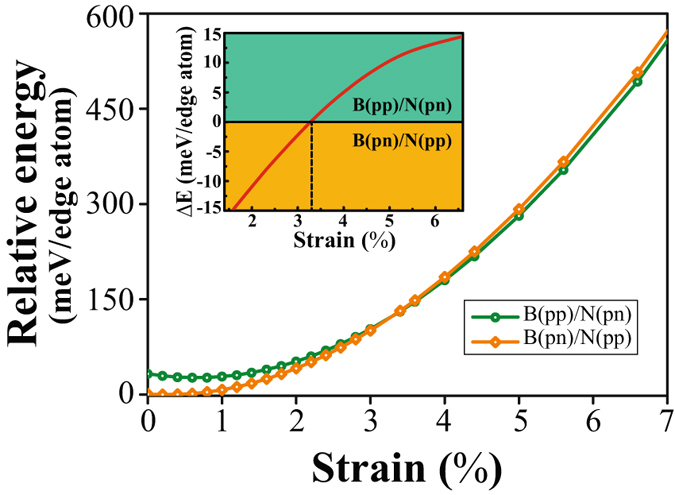
Energy change with uniaxial strain for magnetic zzBNNRs, suggesting the strain-induced transition of magnetic states from B(pn)/N(pp) to B(pp)/N(pn). The inset shows the energy difference between B(pn)/N(pp) and B(pp)/N(pn) with uniaxial strain.

Above results have demonstrated the effects of magnetic coupling on the stress states at the edges of zzBNNRs. On the other hand, it is expected that an uniaxial stress or strain could induce the change of magnetic states of zzBNNRs. In order to investigate the stress/strain induced magnetic transition, we examine the correlation between two representative states, namely B(pn)/N(pp) (the magnetic ground state) and B(pp)/N(pn) (the least energetically favorable state). Figure 6 plots the variation of potential energies of B(pn)/N(pp) and B(pp)/N(pn) as a function of the strain. It is clear from the inset of Fig. 6 that after the tensile strain exceeds around 3.3%, the potential energy of B(pn)/N(pp) is larger than that of B(pp)/N(pn). It indicates that B(pp)/N(pn) becomes more energetically favorable than ground state B(pn)/N(pp). This can be explained by that as the tensile strain increases, larger compressive edge stress of B(pp)/N(pn)) is more favorable to counteract the energy increase due to the stretch of zzBNNRs. When the uniaxial tensile strain reaches a critical value identified by the intersection of two energy curves, the magnetic coupling would undergo a transition from one state to another accordingly. By using the similar method, we have estimated other critical strain values at 3.9% and 20% for magnetic phase transitions from B(pn)/N(pp) ground state to B(pn)/N(pn) and B(pp)/N(pp) metastable states, respectively. It should be noted that 20% strain could not feasible in practice due to bond breaking. These results have demonstrated the capability of strain engineering to programmably control the magnetic properties of zzBNNRs in a flexible manner.

Notably, the edge magnetic configuration also affects the conduction characteristics at the edge of zzBNNR. As shown in Fig. 5, both B-terminated and N-terminated edges with ferromagnetic ordering are in half-metallic behavior, while those with antiferromagnetic ordering are metallic states retaining the same electronic states in both spin-up and spin-down channel. Such half-metallicity obtained at edges with ferromagnetic ordering suggests zzBNNR as a promising material for the metal-free spintronics application^33, 34^. Interestingly, the magnetic phase transitions caused by strain engineering also lead to the metallic to half-metallic phase transitions at edges of zzBNNR correspondingly. It could be an effective way for utilizing magnetic zzBNNR in the tunable spintronic devices.

## Conclusion

In summary, our atomistic simulations have revealed that different magnetic configurations lead to different edge stress states for zigzag BN nanoribbons. These distinct edge stress states caused by magnetic configurations can effectively distort the local bonding environment at specific edges. Further PDOS calculations also indicate that the different magnetic orderings correspond to a rich spectrum of electronic behaviors at edges. By applying an uniaxial tensile strain, we found that the spin arrangements at each edge of zzBNNRs undergo a magnetic transition from B(pn)/N(pp) to B(pp)/N(pn) at the critical strain of 3.3%. Consequently, the change of magnetic states results in metallic 〈-〉 half-metallic transition at B and N edges, respectively. Our findings demonstrate that the magnetic and electronic structure of bare zzBNNRs can be switched by strain engineering, suggesting a key feature in the design of future BN-based spintronic devices.

## Methods

### Computational methods

The bare zzBNNRs with widths (W) ranging from 1.16 nm to 2.90 nm are investigated in present work. The model is constructed using a double periodicity unit cell with two B atoms at one edge and two N atoms at the other edge as shown in Fig. 1. The periodic images were separated by a 16 Å vacuum to avoid any spurious interactions. To obtain optimized geometries and their related energies and electronic structures of all BN nanoribbons considered in our study, spin polarized first-principles DFT calculations were performed using the Vienna ab initio simulation package (VASP)^35^. Electron exchange and correlation were described using the generalized-gradient approximation (GGA) of the Perdew-Burke-Ernzerhof (PBE) form ref. 36 and projected-augmented wave (PAW) potentials were used to treat core and valence potentials with a kinetic energy cut-off of 500 eV for the plane-wave basis set^37^.

### Data availability

All relevant data are available from the authors.
